# Progress in liquid crystal chemistry II

**DOI:** 10.3762/bjoc.8.13

**Published:** 2012-01-24

**Authors:** Sabine Laschat

**Affiliations:** 1 Institut für Organische Chemie, Universität Stuttgart, Pfaffenwaldring 55, D-70569 Stuttgart, Germany

It is a great pleasure to introduce this second Thematic Series on "Progress in liquid crystal chemistry" within the *Beilstein Journal of Organic Chemistry*. Why liquid crystals?

Liquid crystals have a major impact on our modern society, reaching from simple digital calculators to high-resolution flat-panel displays, and their market volume is expected to further increase. For example, in 2010 revenues of €1,013 million from sales of liquid crystals were reported by Merck, the world’s largest supplier of liquid crystal materials for electro-optical applications. This increase of 38% on 2009 was attributed to the demand for liquid crystal displays (LCDs). Forecasts predict annual global shipments of over 268.8 million LCD television sets by 2014 [1]. However, the chemistry behind liquid crystals, and particularly the synthesis, is the platform for such tremendous technological achievements, and this is true for both calamitic (rod-like) as well as discotic (disk-shaped) or other types of liquid crystals [2–4].

Since our initial Thematic Series "Progress in liquid crystal chemistry" two years ago [5], the research field has further broadened and stretches currently from materials science, through energy conversion and storage, to life sciences. The interested reader might be directed to a very recent special issue of *Liquid Crystals* edited by John W. Goodby to find more detailed information [6].

The review articles and original research papers of this second Thematic Series in the *Beilstein Journal of Organic Chemistry*, written by known experts of their field, cover diverse topics such as novel discotic and calamitic compounds, bent-core mesogens, amphiphiles, and liquid-crystalline nanoparticles. It is our goal to give the reader insight into both synthetic aspects of liquid crystal chemistry as well as application-oriented properties, such as photoconductivity or chirality transfer, to name just a few selected examples. Finally, we would particularly like to inspire people with complementary expertise, working in different fields of chemistry, to join forces with the liquid crystal community and to introduce their specific knowledge and objectives in order to aid the further advancement of this topic.

Sabine Laschat

Stuttgart, January 2012
